# DOE Optimization of Nano-based Carrier of Pregabalin as Hydrogel: New Therapeutic & Chemometric Approaches for Controlled Drug Delivery Systems

**DOI:** 10.1038/srep41503

**Published:** 2017-01-30

**Authors:** Mona G. Arafa, Bassam M. Ayoub

**Affiliations:** 1 Pharmaceutics Department, Faculty of Pharmacy, The British University in Egypt (BUE), El-Sherouk city, Cairo 11837, Egypt; 2 The Center for Drug Research and Development (CDRD), The British University in Egypt (BUE), El-Sherouk city, Cairo 11837, Egypt; 3 Chemotheraputic Unit, Mansoura University Hospitals, Mansoura 35516, Egypt; 4 Pharmaceutical Chemistry Department, Faculty of Pharmacy, The British University in Egypt (BUE), El-Sherouk city, Cairo 11837, Egypt

## Abstract

Niosomes entrapping pregabalin (PG) were prepared using span 60 and cholesterol in different molar ratios by hydration method, the remaining PG from the hydrating solution was separated from vesicles by freeze centrifugation. Optimization of nano-based carrier of pregabalin (PG) was achieved. Quality by Design strategy was successfully employed to obtain PG-loaded niosomes with the desired properties. The optimal particle size, drug release and entrapment efficiency were attained by Minitab^®^ program using design of experiment (DOE) that predicted the best parameters by investigating the combined effect of different factors simultaneously. Pareto chart was used in the screening step to exclude the insignificant variables while response surface methodology (RSM) was used in the optimization step to study the significant factors. Best formula was selected to prepare topical hydrogels loaded with niosomal PG using HPMC and Carbopol 934. It was verified, by means of mechanical and rheological tests, that addition of the vesicles to the gel matrix affected significantly gel network. *In vitro* release and *ex vivo* permeation experiments were carried out. Delivery of PG molecules followed a Higuchi, non Fickian diffusion. The present work will be of interest for pharmaceutical industry as a controlled transdermal alternative to the conventional oral route.

Fibromyalgia syndrome is characterized by continuous pain in the body that persists for more than three months^1^. In addition, sciatic neuralgia is a localized leg pain with a sharp burning quality associated with numbness^2^. Furthermore, back pain as a result of nerves compression in the cervical area radiates to the leg^3^. Fibromyalgia with sciatica neuralgia and low back pain are treated by different interventions as oral therapy, trans-dermal or by injection in addition to anti-inflammatory and muscle relaxant drugs^4^. One of the FDA approved drugs for such a treatment is pregabalin (PG)^5^. PG is an alpha-2-delta ligand similar to GABA, binds to subunit of the calcium channels causing a reduction of calcium flow. Consequently, the release of pain neurotransmitters will be inhibited^6^.

Pregabalin has side effects when taken orally such as dizziness, sleepiness, dry mouth, blurred vision, difficulty with concentration, hyper-sensitivity and decreased platelet count. The skin forms an attractive and accessible route of systemic drug delivery because of the avoidance of the previously mentioned side effects associated with oral route of administration. Many approaches have been investigated to enhance the drug permeation through the skin barrier for its use as transdermal drug delivery^7^. Nano-vesicles have attracted a great deal of attention in the transdermal delivery field because of many advantages such as prolonging the therapeutic effect of the drug as they prevent the excess drug from pouring into the systemic circulation and thus reducing adverse reactions with the great possibility to modulate drug bioavailability^8^. All these findings are in consistence with many reported studies of niosomal formulations of different therapeutic agents that showed a gradual increase in their activity due to the controlled release of drug^9^,^10^,^11^. Inclusion of nano-vesicles as niosomes in hydrogels increases drug uptake, decreases skin irritation and avoids first pass effect which may be attributed to the deep access of nanoparticles to the human body because of its particle size and surface properties, in addition to controlling the release of the drug specially when the entrapped drug is hydrophilic^12^,^13^,^14^.

In the present work; inclusion of PG in niosomal hydrogel preparation is of great importance as many reported PG formulations including PG conventional topical gel^15^, PG patch^16^ and PG solution^17^ were not enhanced with such innovative nano-vesicles, resulting in the lacking of their ability to deliver PG in controlled pattern with good skin permeation profiles to achieve the bioavailability augmentation.

In this light; the main aim of the present work was to attain the optimal nano-carrier design of PG including colloidal behavior, intracellular penetration and controlled pharmacokinetic profile. To achieve this novel approach; PG niosomes were prepared by the hydration of a thin film formed of surfactant and cholesterol to obtain niosomal vesicles entrapping drug inside. The remaining PG in the hydration solution was separated from the vesicles by freeze centrifugation that also enhanced the entrapment efficiency of niosomal PG^18^,^19^,^20^. Moreover, formulation of topical PG hydrogels, using HPMC and Carbopol 934 as gel bases was achieved as one of the most effective methodological approaches for the development of a new dosage form. Design of experiment (DOE) was used for the handling of all factors simultaneously while optimization of niosomes to improve the understanding of the significant variables and extract the most useful information. Evaluation of *in vitro* release profile in addition to *ex vivo* investigation of the rat skin permeation ability of PG from the developed niosomal formulations was employed as a reflection of the *in vivo* scenario to ensure its systemic effect in a controlled manner via transdermal route in comparison to hydrogels loaded PG.

## Materials and Methodology

### Instruments

Viscometer HAKE RV3 and digital circulating water bath (Goerzallee 249, Germany), Digital precise shaking water bath (WSB-18, Dahan Scientific Co. Ltd., Korea), Ultraviolet-visible spectrophotometer (V-630, Jasco, Japan), Elmasonic (S60 H, Elma Hans Schmidbauer GmbH, Germany), pH-meter (CA 92634, Beckman Instruments Fullerton, USA), Scanning electron microscope (JEOL 5500 LV., Tokyo, Japan), Transmission electron microscope (JEOL JSM-6510 LV., Tokyo, Japan), X-ray diffractometric (D8, Buruke Co., Germany), Differential scanning calorimeter (DSC-60, Shimadzu, Japan), Rotary evaporator (OSB-2100, N-1200A, Shanghai Eyela Co. Ltd., China), Freeze centrifuge (2-16KL, Sigma Laborzentrifugen GmbH, Germany) and Malvern Instruments Ltd (Zetasizer Nano-Zs90, MPT-Z, UK) were used.

### Materials and reagents

Pharmaceutical grade PG (99.90%) was kindly supplied from Pfizer Inc., U.S.A., Cholesterol (95% stabilized) was purchased from Acros organics, U.K. Span 60, sorbitan stearate, sorbitane monostearate and chloroform were purchased from Sigma-Aldrich, Germany. HydroxyPropylemethylcellulose (HPMC) was kindly supplied from Cairo Pharmaceuticals Co., Egypt. Carbopol 934 was kindly provided by El-Nil Co. Pharmaceuticals & Chemical Industries, Cairo, Egypt. Monopotassium dihydrogen phosphate and sodium phosphate dibasic anhydrous were bought from Adwic, Al Nasr Pharmaceutical Chemicals.co. Egypt. Cellophane membrane; Spectra/por dialysis membrane 12000–14000 Mwt cut off was used.

### Preparation and characterization of Niosomes

#### PG vesicle preparation

Different molar ratios (4:1, 4:4 & 4:7, respectively) of non-ionic surfactant (span 60) and cholesterol were accurately weighed and dissolved in chloroform in a round-bottom flask. Chloroform was evaporated at 60 °C under vacuum and the resulting film was dried under vacuum over night at 25 °C. PG (1.5 gm) was dissolved in the least amount of distilled water and then the previous mixture was used in the hydration step of niosomes vesicles. The obtained film was hydrated by addition of the least amount of distilled water containing PG at 40 °C. The dispersion was vortexed for 5 minutes, sonicated at 55 °C for 30 minutes at 20 kHz using amplitude of 16%. Niosomes dispersion was finally centrifuged at 7000 rpm for one hour using freeze centrifugation at 4 °C to avoid disruption of niosomes^21^,^22^,^23^.

Niosomal pellets were re-suspended in distilled water and then centrifuged again. This washing procedure was repeated to ensure that the un-entrapped PG was no longer present in the void volume between niosomes^23^. Lyophilization of niosomes was carried out by freezing niosomes dispersion at −70 °C and drying under pressure for 24 hours to get niosome powders. All formulae (F1, F2 and F3) are presented in (Table 1).

#### Characterization of niosomes

Scanning electron microscopy (SEM) and transmission electron microscopy (TEM) experiments were conducted to examine the formation of niosomes vesicles, their surface characteristics and morphology, while zetasizer was employed to determine their particles size and size distribution. In addition, thermal and phase transitional temperature behavior of PG and niosomes individual components were examined using differential scanning calorimeter (DSC). X-ray diffractometer (XRD) analysis was used to investigate the crystalline structure of niosomes and PG. Entrapment efficiency calculations (EE %) were applied. *In vitro* drug release and *ex vivo* permeation studies were investigated as a guide for *in vivo* performance of the newly developed dosage form.

#### Scanning electron microscopy (SEM) of niosomes

Samples were sprinkled on SEM holder with double sided adhesive tape, coated with a layer of 150 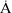 gold for two minutes using a SPI module TM gold sputter coater and examined using a high vacuum mode of SEM^24^.

#### Transmission electron microscopy (TEM) of niosomes

A 10-fold aqueous diluted drop of the niosomal dispersion was subjected to collodion-coated 300 mesh copper grid, left for 5 minutes, adsorbed using filter paper then a drop of 2% aqueous uranyl acetate was applied for 1 minutes, the remaining solution was removed and the samples was air dried and examined at 80 KV^24^.

#### Particle size and size distribution of niosomes

Nanoparticle size distribution was determined using photon correlation spectroscopy (zeta potential analyzer; Malvern Zetasize Nano-zs90, Malvern Instruments, Malvern, UK). The size distribution analysis was performed at a scattering angle of 90 degrees and at a temperature of 25 °C using samples appropriately diluted with dispersant water. For each sample, the mean diameter ± standard deviation of 10 determinations was calculated applying multimodal analysis.

#### Differential scanning calorimetry (DSC) of niosomes

Heating rate of 10 °C/min was employed over a temperature range of 20–250 °C for the niosomal formulations and their individual components with nitrogen purging (50 ml/min). PG powder was investigated over a temperature range of 20–400 °C. The weights used for the niosomal examination were equivalent to 1 mg for each individual component, while 2 mg of PG was employed for drug examination. A slandered aluminum sample pans were used, an empty pan was used^25^.

#### X-Ray diffraction (XRD) of niosomes

Samples were placed in a special plane glass. Small-angle X-ray diffraction was obtained by using an X-ray super speed diffractometer with a Ni filter and Cu radiation (*λ* = 0.542 nm), tube voltage 25–45 kV and tube current 100–200 mA, and scanned from 2° to 70°, 2*θ*^26^.

#### Entrapment efficiency (EE %) of PG in niosomes

0.1 ml of PG suspension was diluted with absolute alcohol and sonicated for ten minutes to obtain a clear solution. Concentration of entrapped PG was determined spectrophotometrically at 210 nm using UV spectrophotometer against the sample withdrawn from empty niosomal dispersion treated in a similar manner. The entrapment efficiency was determined relative to the original drug concentration as (EE % = ED/TD *100, Equation 1) Where EE % is the entrapment efficiency percent, ED is the entrapped drug concentration and TD is the theoretical drug concentration^27^.

#### *In vitro* release of PG from niosomes

One ml of phosphate buffer (pH 7.4) was used to suspend twenty mg of PG formulation. Suspension was placed in a glass tube; its lower end was covered by a soaked cellulose membrane. The glass tube was placed in a beaker containing 50 ml of the same phosphate buffer (maintained at 37 °C) as dissolution medium. The beaker was placed in a water bath under mild agitation (50 rpm). At predetermined time intervals for eight hours, aliquots were withdrawn and replaced with the same volume of fresh buffer and PG content was determined in triplicates by UV spectroscopy at 210 nm. Release profile was subjected to kinetic analysis using linear regression according to zero-order kinetic (C = C_o_ − K_o_t, Equation 2), first-order kinetic (log C = log C_o_sK_1_ t/2.303, Equation 3) and Higuchi diffusion kinetics (Q/A = 2 C_o_ (D/ K_Π_)^½^ t^1/2^, Equation 4)^28^.

### Preparation and characterization of topical gels

Different formulations (F4, F5, F6 and F7) of muco-adhesive topical gels containing PG alone or PG in niosomes (Table 2) were prepared.

### Preparation of plain and niosomal topical gels

Two percent *w/w* of gel bases (HPMC and carbopol 934) was sprinkled gently with continuous stirring on the surface of distilled water. The dispersion was stirred until a clear transparent gel is formed using a magnetic stirrer. Then, 0.5 gm PG was dissolved in the polymer dispersion under constant stirring until uniform and clear solution was obtained. The resultant gel was left over night and then filled in dry glass containers. Triethanolamine (0.1 mL) was added in the stirring step while carbopol 934 gel preparation^29^. PG-loaded niosomes equivalent to 0.5 gm PG were mixed with plain gels under continuous stirring and then method of preparation was adopted^30^.

### Characterization of the prepared gels

#### Determination of PG content in the prepared gels

One gram from each formulation containing PG was accurately weighed and transferred to a closed volumetric flask containing 10 ml methanol, shacked for 15 minutes, diluted to 100 ml with phosphate buffer pH (7.4) and then solution was centrifuged. The supernatant was filtered using Millipore filter and measured spectrophotometrically at 210 nm for PG content^31^.

#### Viscosity determination of the prepared gels

Rheological determination of the prepared gels was determined using Rotary viscometer (con and plate viscometer). One gram of each PG formulation was placed on the viscometer plate with a diameter of 2.9 cm and Roto Cone with 2.8 cm in diameter. The viscosity of samples was measured at different angular velocities at temperature of 37 °C ± 0.5 °C. A typical run involved changing the angular velocity and the reading taken after equal time intervals for each velocity. The angular velocity was reversed and the average of two readings was used to calculate the velocity. The viscosity was measured as (η = G.S/N, Equation 5) where; η is the viscosity in mPa.s (mPa.s = 1 centipoise), G is the instrumental factor equals to 14200 (mPa.s/scalgrade.min), S is the Torque (scale grade) and N is the speed (rpm)^32^.

#### *In vitro* release study of PG from gels

The diffusion cells were thermo-regulated with water bath at 37 °C. The semi-permeable cellophane membranes were soaked in phosphate buffer (pH 7.4), dried and pulled to cover the open end of a glass tube having a diameter of 3 cm and diffusion area equals (7.068 cm^2^) using rubber band. One gram of each formula (equivalent to 5 mg PG) was thoroughly distributed on the membrane to cover the diameter of 3 cm circle. To each tube, 1.5 ml of the same solution was added. The tubes in inverted position were then immersed in a beaker containing preheated 50 mL phosphate buffer of pH 7.4 that maintained at 37 °C. The whole assembly shacked at 25 strokes per minute during the entire time of diffusion. For each gel sample and at predetermined time intervals of 30, 60, 120, 180, 240, 300, 360, 420 and 480 min, aliquots were withdrawn and compensated by the same volumes of freshly prepared medium maintained at the same temperature. The released amounts of PG were analyzed spectrophotometrically at 210 nm^33^.

#### *Ex vivo* permeation study of PG hydrogels

The *ex vivo* permeation of PG from different proposed topical hydrogels was measured using excised skin of rats with average weight of 230–300 grams. All animal work was conducted in accordance with the guidelines outlined in the *Guide for the Care and Use of Laboratory Animals* and was approved by the Ethical Committee of the British University in Egypt. The rats were sacrificed using anesthetic ether, the hair of abdominal skin was removed, the fat adhering to the dermis side was detached and the skin was rinsed with phosphate buffer of pH 7.4. The *ex vivo* permeation through excised skin was performed by Franz diffusion cells that consist of two chambers, the donor and the receptor compartment with an available diffusion area of 2.25 cm^2^. The donor compartment was open at the top and was exposed to atmosphere. The excised skin was mounted between the compartments of the diffusion cell with stratum corneum facing the donor compartment and clamped into position. Magnetic stirrer bars were added to the receptor chambers and filled with the receptor phase. Phosphate buffer of pH 7.4 was used as the receptor medium^34^. The entire setup was placed over magnetic stirrer, and the temperature was maintained at 37 ± 0.5 °C. The skin sections were initially left in the Franz cells for 2 hours in order to facilitate hydration of the skin samples. After that 1 g of each prepared topical gel containing 5 mg PG was applied onto the excised skin fitted on the Franz cell. Serial samples were collected from the receptor compartment at predetermined intervals over the study period and fresh receptor liquid was added to the receptor compartment in order to replace the buffer. The amount of permeated PG was determined by UV-VIS spectrophotometer at 210 nm then the amount of permeated PG was plotted against time. The permeation flux was calculated^35^ using Equation 7 as Jss = dQ/(dt * A) where Jss is the steady-state permeation flux (μg/cm^2^/h), A is the area of skin tissue (cm^2^) through which drug permeation takes place, and (dQ/dt) is the amount of drug passing through the skin per unit time at a steady state.

#### Kinetic release study

*In vitro* release data were analyzed using linear regression according to zero-order kinetic, first-order kinetic and Higuchi diffusion kinetics. Also Korsmeyer–Peppas model was studied (Mt/M_∞_ = kt^n^, Equation 6); where Mt is the amount of drug released at time t, M_∞_ is the amount of drug released as time approaches infinity, k is a constant incorporating characteristics of the particle system or network and n is the diffusion exponent^36^.

#### Statistical analysis

The *in vitro* release of PG from different topical hydrogels and *ex vivo* permeation experiments using rat skin were estimated by ordinary non-linear regression and student paired t- test with p value of < 0.05. The tests were performed using prism 5 computer program (graph pad software Inc., 5 Demo. Ink.).

## Results and Discussion

### Method development using full factorial design (two levels with center points in case of numeric variables)

Design of experiment (DOE) using Minitab^®^ program was implemented in which all factors affecting the three investigated responses were varied together. Literature review and preliminary investigations were used to select the initial variables with the appropriate levels^20^,^22^,^33^,^37^,^38^. Full factorial design was used to determine the variables which have important effect on particle size, drug release and entrapment efficiency using two levels with center points in case of numeric variables (Table 3). A typical noisome vesicle would consist of biodegradable, biocompatible, and non-immunogenic, non-ionic surfactants such as spans or tweens with different HLB values that affects the entrapment efficiency of the drug, which is also stabilized by the addition of the optimum amount of cholesterol to prepare stable vesicles for the entrapment of water soluble substances^20^,^22^,^33^. Further increase of cholesterol content above certain limit reduces the entrapment efficiency due to the disruption of the regular bilayer structure^37^; so DOE levels were set on different molar ratios within those limits to investigate the optimum ratio of cholesterol and surfactant. In addition, we had to use the least amount of water while niosomes preparation as high aqueous solubility of PG may lead to diffusion out from the vesicles to the aqueous phase^38^, so we applied DOE to examine this variable within the lowest possible limits (10 mL–20 mL). Furthermore, DOE was used to deduce the most suitable surfactant as a categorical variable; using such a categorical variable, rather than numerical, excluded some designs such as Box-Behnken design and so full factorial design was selected for the underlying investigation.

**Figure 1:**
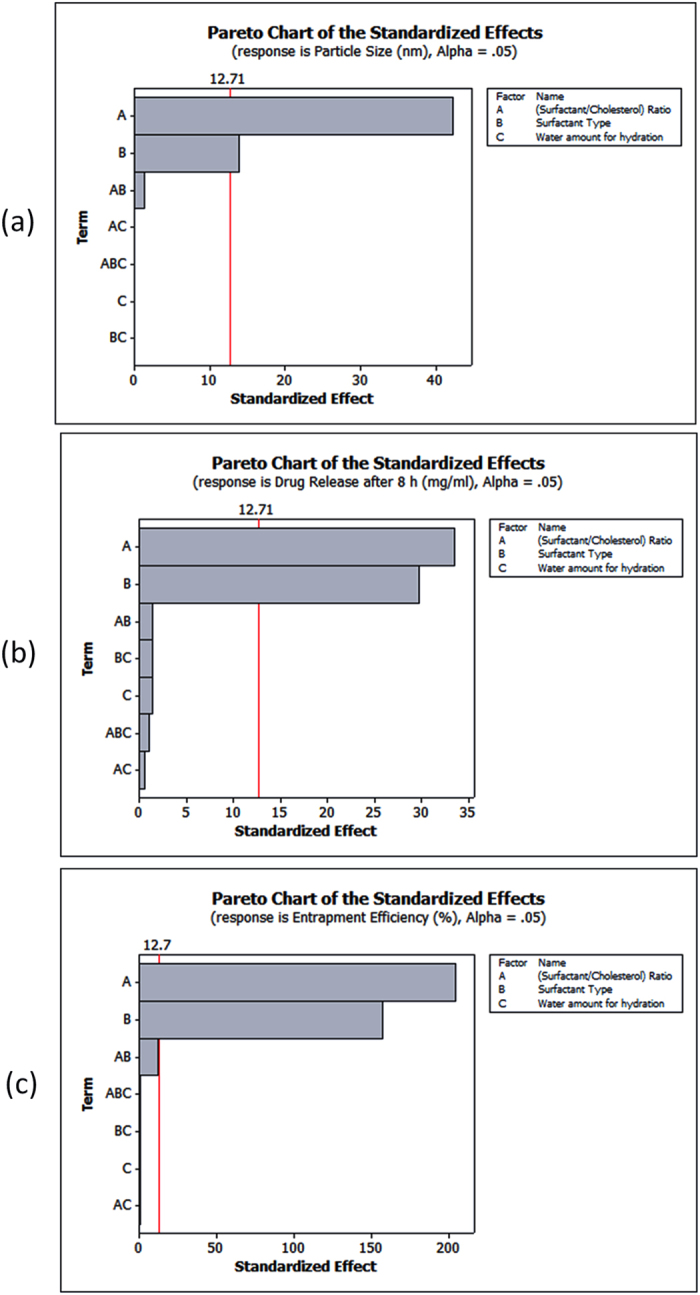
Pareto charts showing the significant variables affecting particle size (**a**), drug release (**b**) and entrapment efficiency (**c**) of PG nanoparticles.

**Figure 2:**
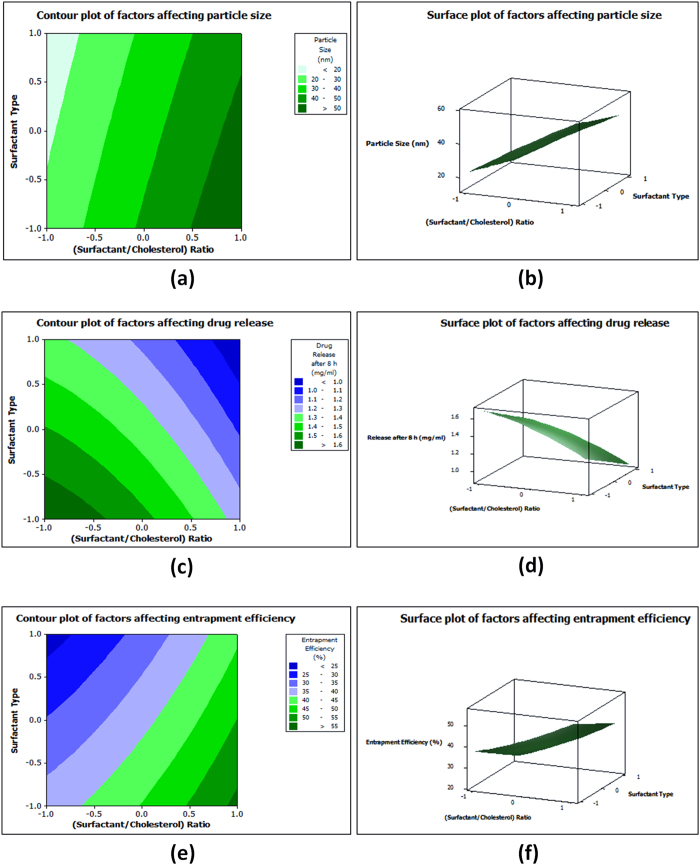
Contour and surface plots of factors affecting particle size (**a**,**b**), drug release (**c**,**d**) and entrapment efficiency (**e**,**f**) of PG nanoparticles.

The studied factors included: (A: surfactant to cholesterol molar ratio) with 4:1 ratio as the low level, 4:7 ratio as the high level and 4:4 ratio as center point of estimation, (B: Surfactant type, Span 60 as -1 and Tween 80 as + 1), (C: Amount of water required for the dry film hydration, 10 mL as −1, 20 mL as + 1 and 15 mL as center point). The factors whose *p*-values were less than 0.05 were considered as “statistically significant”. A graphical display was given as a Pareto chart (Fig. 1) and showed that the variable (C) is statistically non-significant variable so it was excluded from the optimization study based on using 10 mL water for the dry film hydration as the practical lowest possible amount based on the preliminary investigations. Response surface methodology (RSM) was used by Minitab^®^ program showing the effect of the two significant variables as a surface in three-dimensional space (Fig. 2). After interpretation of the developed contour plots and surface plots, it was observed that surfactant type (variable B) was optimum at the low level (−1) which means that Span 60 is better than tween 80 especially regarding drug release and entrapment efficiency. Surfactant: cholesterol molar ratio (variable A) showed the highest entrapment efficiency at the high level (+1) while it showed the highest drug release and lowest particle size at the low level (−1) so the intermediate level (center point of estimation) as 4:4 molar ratio was selected to enhance drug release, entrapment efficiency and particle size parameters in the underlying work.

### Characterization of niosomal vesicles

#### Entrapment efficiency (EE %)

The effect of Span 60: cholesterol molar ratio on the EE % of PG into niosomes is illustrated in (Table 4). The data revealed that EE% of PG increases with each increment of cholesterol ratio. The EE % of PG in niosomes was found to be 37.29 ± 0.48, 45.09 ± 0.18 and 56.49 ± 0.26 for niosomes composed of Span 60 and cholesterol using 4:1, 4:4, and 4:7 molar ratio, respectively. Increasing cholesterol content abolished conversion of the gel of the niosomal system into liquid and thus encapsulation of hydrophilic drugs was enhanced. Cholesterol cemented the leaking space in the bi-layer membranes; consequently the membrane became more firm as surfactants were more condensed while filling^39^. In addition, Entrapment efficiency of PG was affected by the method of free drug separation and freezing the prepared niosomes at −20 °C followed by centrifugation, resulted in a significant increase in PG entrapment inside niosomes. The mechanism explained the events during freeze thawing cycle that the drug and vesicles were concentrated while freezing and particles were closely packed with each other resulted in fusion of niosomal vesicles which entrapped efficiently PG^23^. Finally niosomes prepared from span 60 were superior to those prepared from other surfactants. This can be explained by many facts, span 60 has the highest phase transition temperature^40^, longer saturated alkyl chain produces high entrapment^41^, the longer alkyl chain influences the HLB value of span 60 and the lower HLB of it gives the highest entrapment efficiency of drug and stability of niosomes^28^.

#### Particle size and size distribution of niosomes

**Figure 3:**
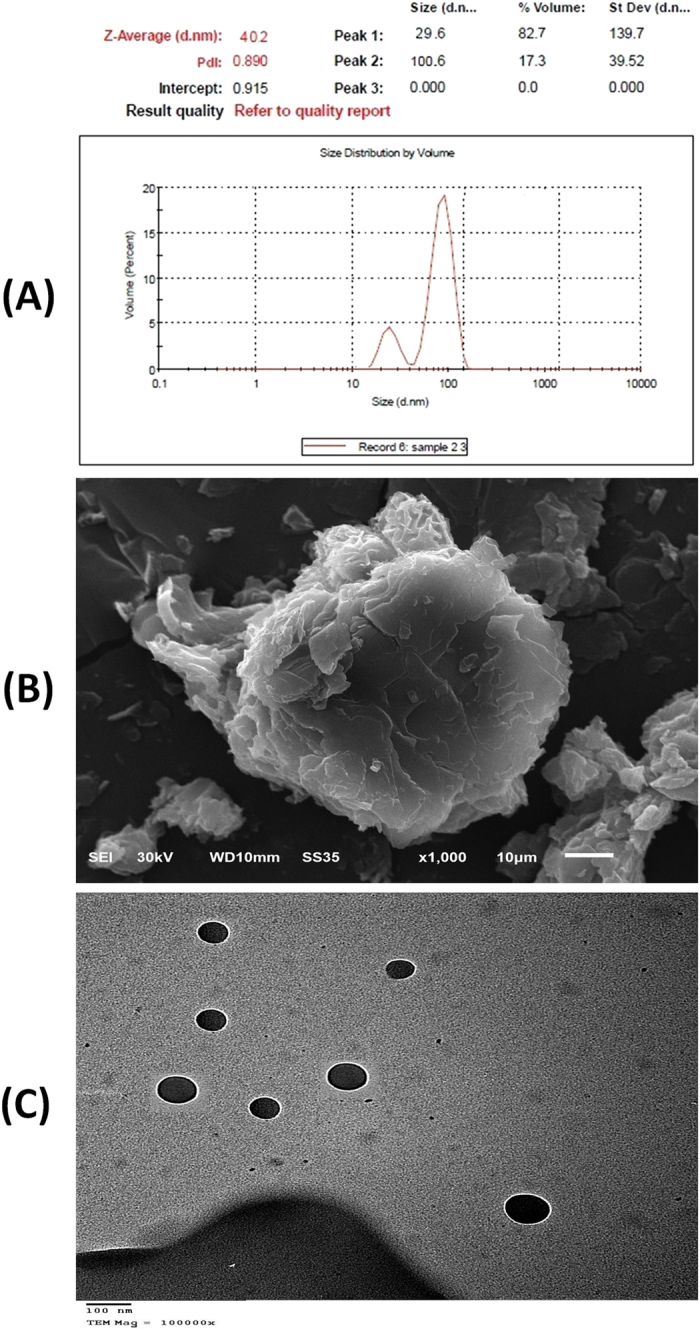
particle size and particle size distribution (**a**) Scanning electron microscopy (**b**) and Transmission electron microscopy (**c**) of formed PG niosomes.

A particle size ranged from 29–100 nm (Fig. 3a) with a dispersant RI 1.330 with viscosity (cP) equals 0.8872 was considered optimum for topical application.

#### Scanning electron microscopy (SEM)

SEM illustrated vesicle diameter and surface characteristics of PG-loaded niosomes (Fig. 3b) which appeared to be smooth due to the filling effect of the surfactant in addition to their thicker layers deposition at deeper invaginations. After dissolution of all components in the solvent, then when solvent evaporated, they crystallized on top of the new surface; that may cause some of the fine crystalline structures to disappear, creating niosomes with smooth surface^42^.

#### Transmission electron mJicroscopy (TEM)

The morphology of the selected formula of PG-loaded niosomes was investigated (Fig. 3c). Vesicles diameter of 29–100 nm revealed the presence of well identified spherical niosomes existing in disperse pattern.

#### X-ray diffractometry (XRD)

**Figure 4:**
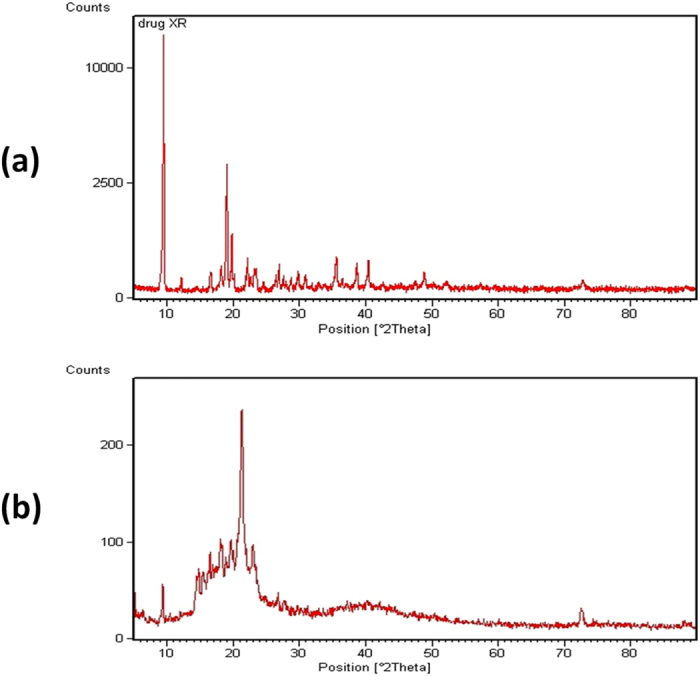
X-ray diffractometry of drug alone (**a**) and formed PG niosomes (**b**).

Crystalline structure of PG and PG-loaded niosomes was investigated (Fig. 4a,b). XRD pattern of PG in its pure form showed that; the main thermal characteristic sharp peaks of PG crystalline form were recorded between 5.8, 18.4, 19.2, 20.7 & 23.7° 2θ. This result was in agreement with a reported study done by (Arnohime, *et al*.)^43^. After the formation of PG-loaded niosomes, it was clearly that PG characteristic peaks were modified in the same thermal events suggesting a partial amorphism. This was in accordance with results reported by (Jana *et al*.)^44^. This also was in agreement with our results obtained under SEM that illustrated smooth surface characteristics of PG-loaded niosomes due to the partial change of crystalline nature of PG^42^. In addition, this partial amorphism may lead to the increment of the entrapment efficiency of PG similar to a previous study done by (Sezgin-Bayindir *et al*.)^45^. It is noticed that these two XRD patterns (bare PG & PG-loaded niosomes) are different in some specific areas as the vesicle formation indeed has effect on the crystalline structure.

#### Differential scanning calorimetry (DSC) of niosomes

**Figure 5:**
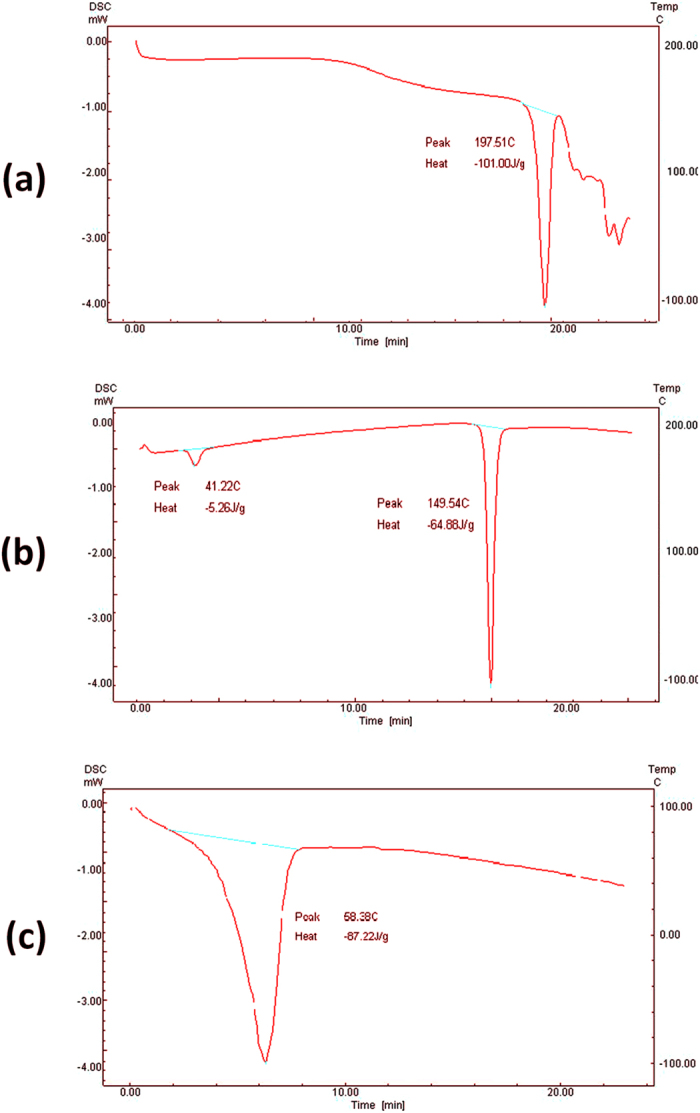
Differential scanning calorimetry of individual components of niosomes including drug (**a**), cholesterol (**b**) and span 60 (**c**).

**Figure 6:**
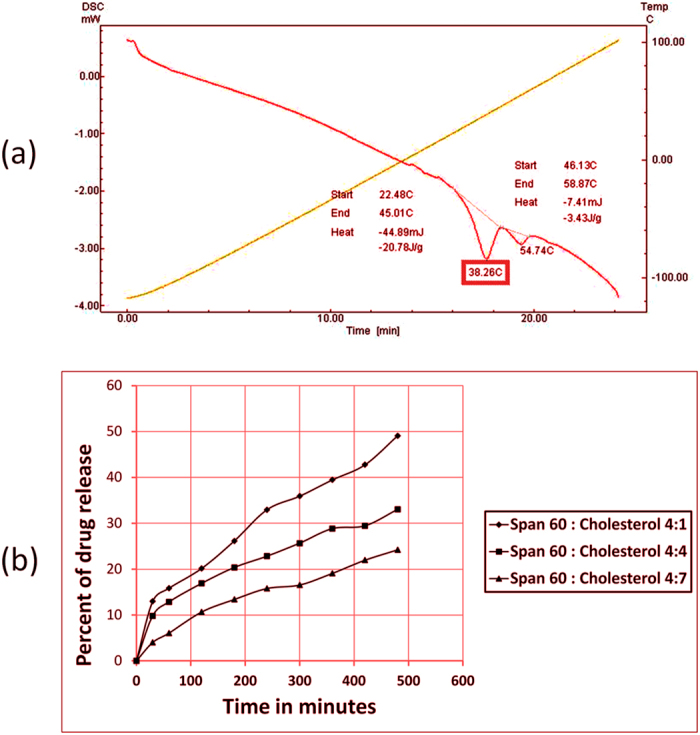
Differential scanning calorimetry of formed PG niosomes prepared using Span 60: cholesterol in molar ratio of 4:4 (**a**) and Release of PG from niosomal vesicles prepared using span 60 and cholesterol in the molar ratios of 4:1, 4:4 and 4:7 in phosphate buffer (pH 7.4) at 37 °C (**b**).

The thermal and phase transitional temperature behavior of PG powder and for the dehydrated pellets of the niosomal formulation were examined. Thermograms (Figs 5a,b,c and 6a) showed a main sharp endothermic peak of PG and cholesterol at 197.5 °C and 149.54 °C, respectively. An endothermic peak of span 60 was observed at 58.38 °C. A change of transition temperature to 38.26 °C was observed for the span 60 and cholesterol in 4:4 molar ratios with some broadening of the endothermic peaks (Fig. 6a). The described thermal events confirmed transition of gel to liquid crystalline state in niosomes representing phase changes when vesicles are formed^46^.

#### *In vitro* release profile of niosomal PG

The percentage released of PG niosomal vesicles, composed of span 60 and cholesterol prepared in different molar ratios of 4:1, 4:4 and 4:7, respectively, was investigated (Fig. 6b). The results showed that, formula three with the highest cholesterol content showed the lowest percent of drug release compared to formulae one and two. PG release percent after eight hours from niosomes composed of span 60 and cholesterol 4:1, 4:4 and 4:7 molar ratios were found to be 48.08 ± 0.93%, 33.02 ± 0.52% and 24.22 ± 0.21% respectively, as by increasing cholesterol content in noisome, the less amount released of drug from the vesicles.

**Figure 7:**
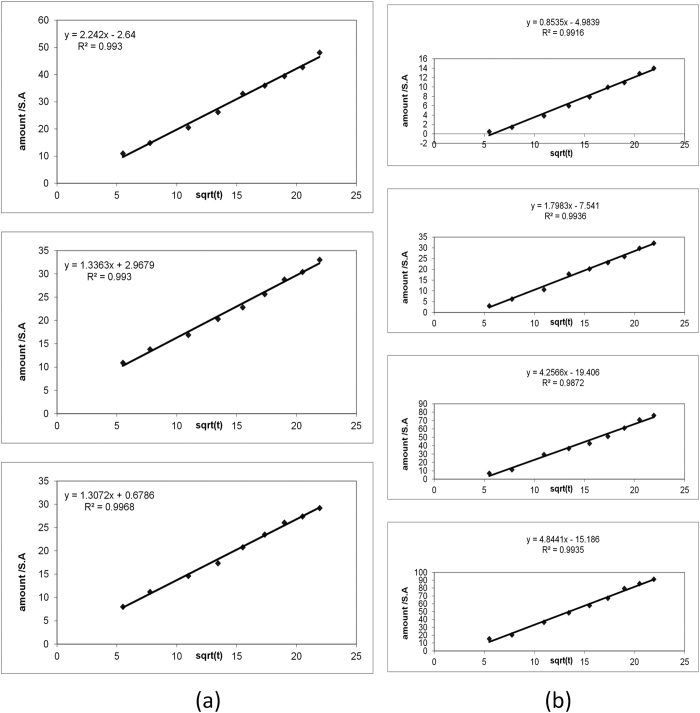
(**a**) Higuchi model of PG niosomes prepared using span 60 and cholesterol in the molar ratios of 4:1 (**a**), 4:4 (**ii**) and 4:7 (**iii**). (**b**) Higuchi diffusion model for PG release from different hydrogel bases namely niosomal carbopol (**i**), niosomal HPMC (**ii**), carbopol (**iii**) and HPMC (**iv**).

Correlation of release data in (Table 5) and (Fig. 7a) revealed that profile is well linearized by Higuchi model^47^ with regression parameters of r^2^ = 0.993, 0.993 and 0.996 for 4:1, 4:4 and 4:7 molar ratios, respectively. This kinetic pattern indicated that release is dominated by diffusion model which normally depends on drug concentration gradient between nano-vesicles and dissolution media with penetration of this media through a porous wall. It means that release can be changed by varying system surface area and wettability, determined by size and uniformity. Mean-while, loading percentage directly affects the drug concentration gradient and release rate.

### Evaluation of PG gels

#### PG content in prepared formulations

The actual PG content in all formulae was illustrated in (Table 6). Gels prepared were containing 1 g of drug that represents percents from 98.32% to 101.12% which is acceptable for all the formulations indicating uniform distribution of the drug and this complies with the official requirements of U.S.P Pharmacopeia^48^.

#### Viscosity of the prepared formulations

**Figure 8:**
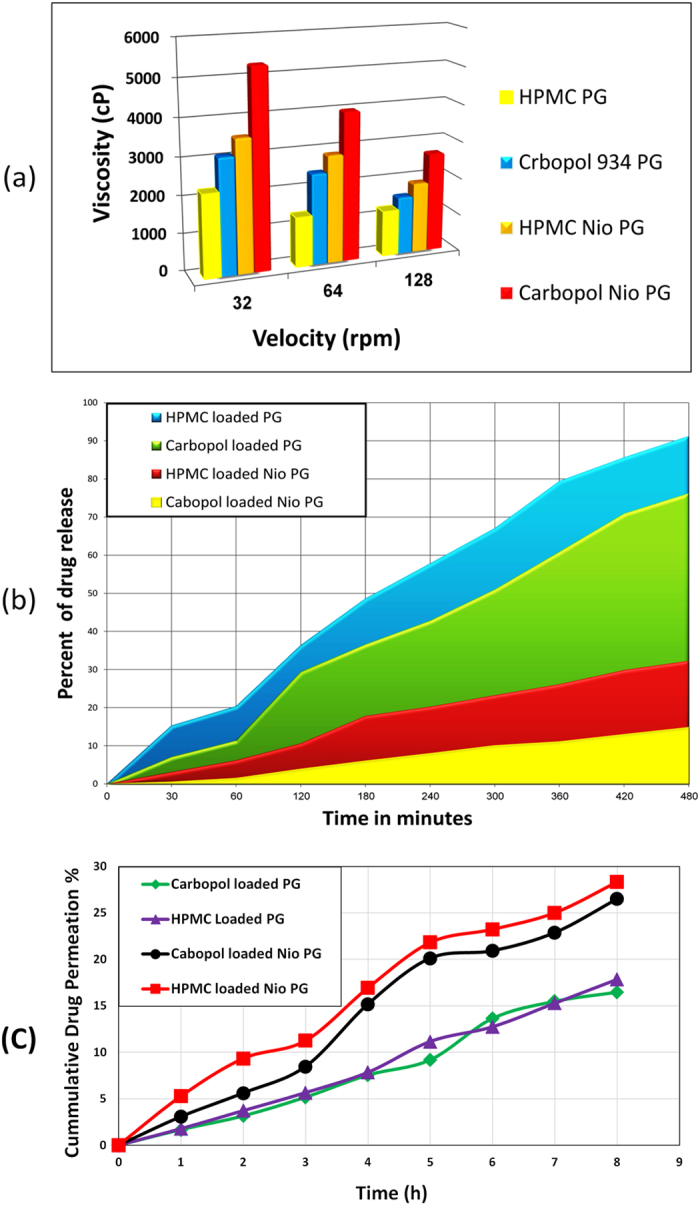
(**a**) Viscosity profile of different PG hydrogels at 32, 64 & 128 rpm. (**b**) Release of different PG gel formulae from HPMC and Carbopol 934 in phosphate buffer (pH 7.4) at 37 °C. (**C**) Permeation studies through excised rats’ skin by HPMC loaded PG, Carbopol loaded PG, HPMC loaded PG niosomes and Carbopol loaded PG niosomes with the same drug concentration (0.5% *w/w*).

Rheological measurements were useful for the characterization of the visco-elastic properties of aqueous PG hydrogels dispersion and niosomal PG hydrogels. Continuous shear investigations were performed in the tested hydrogel formulations in order to evaluate the shear rate as a function of shear stress^42^ as the rheological behavior of the prepared gels is important in drug release. The rheological profiles of all gelling systems of PG are shown in (Table 6) and (Fig. 8). Viscosity was in the following order: F7 > F6 > F5 > F4 which were 5325 > 3550 > 3106.25 > 2218.75 cP., respectively at 32 rpm (Table 5) as incorporating gel in solid lipid resulted in a significant increase of viscous characters of gels.

These results may be attributed to the interactions between gel forming polymer and lipids which can affect the semisolid consistency of the transdermal formulations and therefore, their rheological status. In case of solid lipids, they tend to align with increasing shear stress alleviating the flow^49^. Furthermore, data illustrated in (Table 6) and (Fig. 8a) also showed that, viscosity of carbopol 934 gels is higher than HPMC gels for all preparations as the high viscosity of carbopol 934 results from the fast and swelling behavior of carbopol 934. On the contrary, HPMC is nonionic hydrophilic polymer with lower viscosity. In addition, when velocity (rate of shear) increases; viscosity decreases for all formulations. For example; viscosity was 2884.38 cP & 1331.25 cP at 64 rpm, meanwhile; it was 1885.94 cP &1220.31 cP at 128 rpm for HPMC niosomal PG and HPMC PG, respectively as increasing shear rate results in decreasing shear stress consequently, decreasing viscosity.

#### *In vitro* release of PG from different gel bases

Drug release profiles of PG hydrogel, and niosomal PG in different gelling agents are presented in (Fig. 8). HPMC hydrogel formulations showed higher release rate (91.2** ± **0.05%) of PG compared to niosomal PG formulations (32.2 ± 0.02%). While carbopol 934 hydrogel formulations showed higher release rate (76.2** ± **0.13%) of PG compared to niosomal PG formulations (14.72** ± **0.21%) as encapsulation of the drug into niosomes resulted in prolonged drug release rate. Furthermore, for niosomal PG gels; the solid matrix of the niosomal PG was responsible for drug immobilization and subsequent lower drug release than hydrogel PG^50^. With the fact that niosomes encapsulation sustains PG release from gels implies that niosomes integrity is not affected by the gel in which niosomes are dispersed^51^. Moreover, drug release patterns confirmed that the type of gelling agent affects the release rate. The amount of PG released from HPMC and carbopol 934 hydrogel after eight hours were 91.2** ± **0.05% and 76.2 ± 0.13% respectively, while those released from HPMC and carbopol 934 niosomal PG gels were 32.2** ± **0.02% and 14.72 ± 0.21% respectively (Fig. 8b). It is obvious that, the amounts of PG released from HPMC are always higher than those released from carbopol 934 at the same time period due to higher viscosity of carbopol 934, its faster rate of swelling, its lower diffusion through the swollen gel layer and faster rate of polymer relaxation.

Kinetic models of different equations were used to mathematically analyze the *in vitro* release pattern of PG to explain the release kinetics of the drug from different transdermal preparations. The used kinetic models were; zero-order, first-order, Higuchi models^52^,^53^. The linearity was evaluated by calculating the linear correlation coefficient (r^2^). The best fit with the highest correlation coefficient (r^2^) was shown with Higuchi model (0.987–0.993) for control hydrogels and (0.991–0.993) for niosomal hydrogels (Table 7 and Fig. 7b) as the release kinetics of some drugs from different hydrogels in the literature followed the Higuchi diffusion model^54^,^55^. In addition, release data were analyzed using Peppas and Korsmeyer equation^56^.

All the examined formulae exhibited n values between 0.5-1 (Table 7) indicating a non-Fickian transport mechanism due to gel erosion and diffusion mechanism.

#### Statistical analysis of release data

Statistical analysis data showed significant difference between F4, F5, F6 and F7 at P < 0.05 and df = 3.

#### *Ex vivo* release of PG from different gel bases

PG permeation from niosomal gels (F6 & F7) as well as control gels (F4 & F5) was found to be sustained over a period of 8 hours (Fig. 8c). Meanwhile, niosomal formulations of PG (F6 & F7) were able to significantly improve (p < 0.05) PG permeation, by 11% & 10% increase, respectively through the rat skin in comparison to their control conventional hydrogels as lipid structure of niosomes enhances the drug permeability through skin^57^. F7 showed the maximum drug permeation of (28.34%) over a period of 8 h, followed by F6 (26.5%), F5 (17.84%) and F4 (16.48%). The permeation flux was calculated for the prepared HPMC loaded PG niosomes and Carbopol loaded PG niosomes and it was found to be 117.41 μg.cm^−2^.h^−1^ & 60.28 μg.cm^−2^.h^−1^, respectively which was significantly higher (p < 0.05) than that of both HPMC loaded PG and Carbopol loaded PG (48.28 μg.cm^−2^.h^−1^ & 45.55 μg.cm^−2^.h^−1^).

The data indicate the permeation enhancing potential of niosomal PG in hydrogels. In accordance with our findings, a new hydrogels loaded PG niosomes ensured drug permeation through rat skin. The obtained results were in agreement with a study done by (Rita Muzzalupo *et al*.)^57^ who reported that the direct contact between vesicles and skin is essential for efficient delivery because of the inclusion of non-ionic surfactant in their structure. Surfactants contribute to the overall penetration enhancement of compounds primarily by adsorption at interfaces, by interacting with biological membranes and by alteration of stratum corneum barrier function as result of reversible lipid modification^58^. This ambiguous behavior could be due to the chemical structure of the surfactant and the differences in the hydrophilic/lipophilic balance^59^.

## Conclusion

Novel Pregabalin niosomes for controlled transdermal drug delivery systems were developed successfully based on innovative HPMC and Carbopol 934 gels using DOE by Minitab^®^ Program as screening and optimization steps. The physicochemical properties and *in vitro* drug release of PG loaded niosomes, PG niosomal hydrogels and PG hydrogels as control matrices have been studied. PG niosomes were prepared using span 60 & cholesterol in different molar ratios and characterized by SEM, TEM, XRD and DSC analysis. It was verified by means of mechanical and rheological tests that the addition of the vesicles to the gel matrix affects significantly gel network. All formulae showed controlled release pattern. Furthermore, *in vitro* release results demonstrated that the delivery of PG followed a Higuchi, non Fickian diffusion. Moreover, the *ex vivo* permeation of PG niosomes was significantly enhanced when compared to conventional PG hydrogels. In conclusion, only hydrogels loaded PG niosomes could act as effective transdermal drug delivery systems with controlled release profiles. The findings achieved in the presented work create a paradigm for future studies to benefit nanotechnology in therapeutic fields which may represent an urge in the development of superior drug delivery systems.

## Additional Information

**How to cite this article**: Arafa, M. G. and Ayoub, B. M. DOE Optimization of Nano-based Carrier of Pregabalin as Hydrogel: New Therapeutic & Chemometric Approaches for Controlled Drug Delivery Systems. *Sci. Rep.* **7**, 41503; doi: 10.1038/srep41503 (2017).

**Publisher's note:** Springer Nature remains neutral with regard to jurisdictional claims in published maps and institutional affiliations.

## Figures and Tables

**Table 1:** Composition of PG niosomes in different molar ratios.

Item	PG	F1 Span 60 : cholesterol, 4:1	F2 Span 60 : cholesterol, 4:4	F3 Span 60 : cholesterol, 4:7	Final form
%	20	42.40 : 37.60	64.8 : 15.2	30.93 : 49.07	Lyophilized powder
g	1.5	3.18 : 2.82	4.86 : 1.14	2.32 : 3.68	

**Table 2:** Composition of different PG gel formulations (0.5% *w/w*).

Formula	Ingredients				
	HPMC (grams)	Carbopol 934 (grams)	Niosomes (grams)	Triethnolamine (mL)	Distilled water (grams)
F4	2	—	—	—	100
F5	—	2	—	0.1	100
F6	2	—	5	—	100
F7	—	2	5	0.1	100

**Table 3:** Variables, levels of variables and responses studied by full factorial design.

Variables			Response		
(Surfactant/Cholesterol) molar ratio	Surfactant: Span 60 (−1) Tween 80 (+1)	Water required for dry film hydration: 10 mL (−1) & 20 mL (+1)	Particle size (nm)	Drug Release (mg/mL) after 8 h	Entrapment Efficiency (%)
4:1 Low level (−1)	−1	−1	23.01	1.68	37.64
		+1	22.34	1.70	36.83
	+1	−1	13.40	1.33	22.97
		+1	14.07	1.29	23.64
4:4 (Center point)	−1	15 ml (Center point)	40.80	1.58	45.18
	+1	15 ml (Center point)	32.17	1.12	31.86
4:7 High level (+1)	−1	−1	58.83	1.28	56.23
		+1	59.12	1.21	56.87
	+1	−1	47.15	0.97	43.71
		+1	46.33	1.01	42.98

**Table 4:** Effect of span 60: cholesterol molar ratio on the entrapment efficiency of PG into niosomes.

Formula	PG (g)	Span 60: cholesterol (molar ratios)	Entrapment efficiency
F1	1.5	4:1	37.29 ± 0.48%
F2	1.5	4:4	45.09 ± 0.18%
F3	1.5	4:7	56.49 ± 0.26%

**Table 5:** *In vitro* release kinetics of PG from different niosomal formulations.

Correlation coefficient r^2^			
Formula	Zero-order	First-order	Higuchi diffusion
F1	0.984	0.957	0.993
F2	0.990	0.989	0.993
F3	0.977	0.985	0.996

**Table 6:** Physical evaluation of different transdermal gels containing PG (5 mg).

Formula	Drug content	η at N = 32 rpm
F4	99.03 ± 0.35	2218.75
F5	98.32 ± 0.93	3106.25
F6	100.41 ± 0.27	3550
F7	101.12 ± 0.15	5325

**Table 7:** The kinetic release profiles of all gelling systems of PG.

Correlation coefficient r^2^				Korsmeyer-Peppas model			Main Transport mechanism
Formula	Zero order	First order	Higuchi diffusion	r^2^	n	k	
F4	0.984	0.966	0.993	0.994	0.676	0.153	Non Fickian
F5	0.984	0.978	0.987	0.986	0.869	0.427	Non Fickian
F6	0.975	0.986	0.993	0.991	0.840	0.716	Non Fickian
F7	0.989	0.990	0.991	0.985	0.913	0.749	Non Fickian
